# The Positive Effects of Priming Exercise on Oxygen Uptake Kinetics and High-Intensity Exercise Performance Are Not Magnified by a Fast-Start Pacing Strategy in Trained Cyclists

**DOI:** 10.1371/journal.pone.0095202

**Published:** 2014-04-16

**Authors:** Renato Aparecido Corrêa Caritá, Camila Coelho Greco, Benedito Sérgio Denadai

**Affiliations:** Human Performance Laboratory, IB – UNESP, Rio Claro, São Paulo, Brazil; University of Barcelona, Faculty of Biology, Spain

## Abstract

The purpose of this study was to determine both the independent and additive effects of prior heavy-intensity exercise and pacing strategies on the VO2 kinetics and performance during high-intensity exercise. Fourteen endurance cyclists (VO_2_max  = 62.8±8.5 mL.kg^−1^.min^−1^) volunteered to participate in the present study with the following protocols: 1) incremental test to determine lactate threshold and VO_2_max; 2) four maximal constant-load tests to estimate critical power; 3) six bouts of exercise, using a fast-start (FS), even-start (ES) or slow-start (SS) pacing strategy, with and without a preceding heavy-intensity exercise session (i.e., 90% critical power). In all conditions, the subjects completed an all-out sprint during the final 60 s of the test as a measure of the performance. For the control condition, the mean response time was significantly shorter (p<0.001) for FS (27±4 s) than for ES (32±5 s) and SS (32±6 s). After the prior exercise, the mean response time was not significantly different among the paced conditions (FS = 24±5 s; ES = 25±5 s; SS = 26±5 s). The end-sprint performance (i.e., mean power output) was only improved (∼3.2%, p<0.01) by prior exercise. Thus, in trained endurance cyclists, an FS pacing strategy does not magnify the positive effects of priming exercise on the overall VO2 kinetics and short-term high-intensity performance.

## Introduction

Exercise intensity domains (i.e., moderate, heavy and severe) are defined according to the blood lactate and oxygen uptake (VO_2_) responses obtained during constant-work-rate exercise [1]. Critical power (CP – the asymptote of the power-time relationship) is considered the lower boundary of the severe-intensity domain [2]. Indeed, during constant-work-rate exercise performed within the severe domain, the VO_2_ rises inexorably (as the slow component of the VO_2_ kinetics increases) to the maximal oxygen uptake (VO_2_max). Exercise tolerance within the severe domain can be predicted and is defined by the curvature constant of the power–time relationship (W′) [3]. Several lines of evidence indicate that the interaction between VO_2_ kinetics, W' and the attainment of VO_2_max can contribute to exercise intolerance during exercise performed in the severe-intensity domain [4]. Some interventions (e.g., pacing, priming exercise and nitrate supplementation) that are used to improve VO_2_ kinetics (i.e., τ – the time taken to reach 63% of the increase in VO_2_ above baseline and/or the slow component of VO_2_ kinetics) can reduce the W' utilization during the initial phase of exercise, improving performance [5] and exercise tolerance [6] during severe-intensity exercise.

Pacing strategy (i.e., the pattern of the rate of energy expenditure) has important effects on exercise tolerance [7] and performance [5]. The self-selected pacing strategy adopted during a time trial is controlled by a complex regulatory system, in which integrated neural control regulates exercise intensity to prevent homeostatic disturbances that might cause injury [8]. Factors such as exercise modality, event duration and performance level can influence the self-selected pacing strategy [9], [10]. Some studies have demonstrated that a fast-start pacing strategy has a positive effect on performance during sports events of up to approximately 2–3 min in duration [11], [12]; in these events, energy is provided by both aerobic and anaerobic pathways [13]. In these conditions, the VO_2_ kinetics is significantly faster, sparing W' utilization during the initial phase of exercise [5], [7]. Interestingly, Jones et al. [7] found that the percentage reduction in the mean response time of VO_2_ was significantly correlated (r = 0.85, p<0.05) with the percentage improvement in exercise tolerance when a fast start was compared with an even-paced exercise.

Warm-up exercise has been extensively performed by athletes before their participation in subsequent vigorous exercise. Indeed, priming exercise performed at heavy or severe intensities domain can improve exercise tolerance during severe-intensity exercise (submaximal and perimaximal) [6], [14]. These positive alterations have been attributed, at least in part, to enhancement of the overall VO_2_ kinetics [6], [15]. Gerbino et al. [16] and MacDonald et al. [17] demonstrated that prior heavy exercise accelerated the monoexponential kinetics (i.e., mean response time) during a second bout of heavy exercise performed 6 min after the first bout. Later, studies using a more comprehensive model (two or three components) to analyze VO_2_ kinetics [6], [18] demonstrated that this overall acceleration could be attributed to the increased amplitude of the primary component and the reduced amplitude of the slow component, with the time constant of the primary component (i.e., τ) remaining unaffected. A similar response (i.e., increased amplitude of the primary component and unchanged τ) was found for exercise that was performed at perimaximal intensities (100%, 110% and 120% of VO_2_max) after prior heavy exercise [14].

Thus, the interventions discussed above (i.e., priming exercise and pacing) seem to have different effects on the VO_2_ kinetics during severe-intensity exercise. The mechanism that underpins these effects is unclear. However, it is possible that different factors contribute to the VO_2_ response profile under these conditions (pacing *vs*. priming exercise). Priming exercise seems to increase blood flow, oxygenation, oxidative enzyme activity and electromyographic activity, thus accelerating the overall VO_2_ response to severe exercise [15]. A positive pacing strategy, which causes a higher initial rate of muscle ATP hydrolysis, can magnify the VO_2_ “error signal”, i.e., the difference between the instantaneous supply and the required rates of oxidative phosphorylation [5]. The absolute rate at which VO_2_ increases after the onset of exercise is a positive function of the “error signal” [19]; therefore, a fast-start pacing strategy results in faster VO_2_ kinetics [5], [7]. Given this scenario, it is possible that priming exercise can amplify the positive effect of a fast-start pacing strategy on VO_2_ kinetics and exercise tolerance/performance during high-intensity exercise. However, the possible additive effects of priming exercise and pacing strategy on these variables are unknown.

The purpose of this study was to determine the independent and additive effects of prior heavy-intensity exercise and pacing strategies on VO_2_ kinetics and performance during high-intensity exercise. The following hypotheses were proposed: 1) A fast-start pacing strategy would shorten the mean response time, and increase peak power output and mean power output during short-term high-intensity exercise; and 2) Priming exercise would shorten the mean response time, and increase peak power output and mean power output during short-term high-intensity exercise irrespectively of the utilized pacing strategy.

## Materials and Methods

### Ethics statement

The present study was approved by the Ethics Committee of the Biosciences Institute – Rio Claro of São Paulo State University, and all subjects provided written informed consent prior to participation. The study was performed in accordance with the declaration of Helsinki.

### Subjects

Fourteen endurance cyclists (26±5 years; 71±9 kg; 175±8 cm) with at least 5 years of experience in the modality volunteered to participate in the present study; these athletes were competing in regional- to national-level meets. The subjects were familiar with the laboratory testing procedures, as they were previously involved in similar evaluations. The subjects were instructed to be fully rested and hydrated at least 3 h postprandially when reporting to the laboratory and to refrain from using caffeine-containing food or beverages, drugs, alcohol, cigarette, or any form of nicotine 24 h before testing. Each subject was tested in a climate-controlled (21–22°C) laboratory at the same time of day (±2 h) to minimize the effects of diurnal biological variation.

### Experimental design

The subjects were required to visit the laboratory on 11 different occasions, separated by at least 24 h, within a period of three weeks. The first visit to the laboratory was to undergo an incremental test to determine the lactate threshold, VO_2_max and the power output at VO_2_max (PVO_2_max). On the following four visits, the subjects underwent four constant-load tests (75%, 80%, 85% and 100% of PVO_2_max) to exhaustion, in random order, to determine the parameters of the power-duration relationship (i.e., CP and W′). The CP model was used to estimate the workload that would be expected to lead to exhaustion in 3 min (P3-min). From the 6^th^ to the 11^th^ visit, the subjects performed three different pacing strategies (fast start, even start, and slow start) with and without a preceding heavy-intensity exercise session.

### Incremental protocol

The incremental protocol was performed on a cycle ergometer (Lode Excalibur Sport, Lode BC, Groningen, Netherlands) with the subjects pedaling at a constant self-selected pedal rate (between 70 and 90 rpm). The chosen pedal rate along with saddle and handle bar height and configuration was recorded and reproduced in subsequent tests. The initial power output was 120 W for 3 min and was then increased by 20 W every 3 min. Capillary blood samples were collected within the final 20 s of each stage for the determination of the blood lactate concentration ([La]). The [La] were determined (YSI 2300, Yellow Springs, Ohio, USA) immediately and the test was stopped when the [La] rose above 4 mM. Plots of [La] against the power output were provided by two independent reviewers, who determined the lactate threshold as the first sudden and sustained increase in blood lactate above resting concentrations [20]. After a rest period of 30 min, the participants performed a fast ramp test. The test began with an initial 5 min of cycling at 25 W below their previously determined lactate threshold, and the power was subsequently increased by 5 W every 12 s until voluntary exhaustion. The protocol was terminated when a drop of more than 5 rpm of their self-selected cadence occurred for more than 5 seconds despite strong verbal encouragement. VO_2_max was defined as the highest average 15-s VO_2_ value recorded during the incremental test. Pulmonary gas exchange was measured continuously using a breath-by-breath analyzer (Cosmed Quark PFTergo, Rome, Italy). Before each test, the O_2_ and CO_2_ analysis systems were calibrated using ambient air and a gas of known O_2_ and CO_2_ concentration according to the manufacturer's instructions, while the gas analyzer turbine flowmeter was calibrated using a 3-L syringe. The heart rate was also monitored throughout the tests (Polar, Kempele, Finland). The PVO_2_max was defined as the power output at which VO_2_max occurred. The work rate that would require 50%Δ (work rate at the lactate threshold plus 50% of the difference between the work rate at the lactate threshold and VO_2_max) was subsequently calculated.

### Determination of the power–duration relationship

The exercise protocol began with a 10 min warm-up at lactate threshold, followed by 5 min of rest prior to the commencement of the exhaustive trial [21]. Thereafter, the subjects exercised for 3 min at 20 W followed by a constant-workload test (75%, 80%, 85% and 100% of PVO_2_max) to voluntary exhaustion or until the subject could not maintain the required cadence (i.e., a cadence <5 rpm of the preferred cadence) despite verbal encouragement [21]. These tests were conducted at the same cadence as the incremental test. During these testing sessions, the participants were not informed of the imposed work rate, their performance times or their heart rate. The exercise tolerance (tlim) was measured to the nearest second. The three equivalents of the 2-parameter model [P = (W′/tlim)+CP; tlim  = W′/(P-CP); W = CP·tlim+W′] were used to fit the data and estimate CP and W′ [22] using an iterative nonlinear regression procedure (Microcal Origin 7.5; Northampton, MA, USA) for each subject. The CP and W′ estimates from the 3 equations were compared to select the best fit using the model associated with the lowest standard error for CP (SEE) [23], [24].

### Experimental sessions

**Figure 1:**
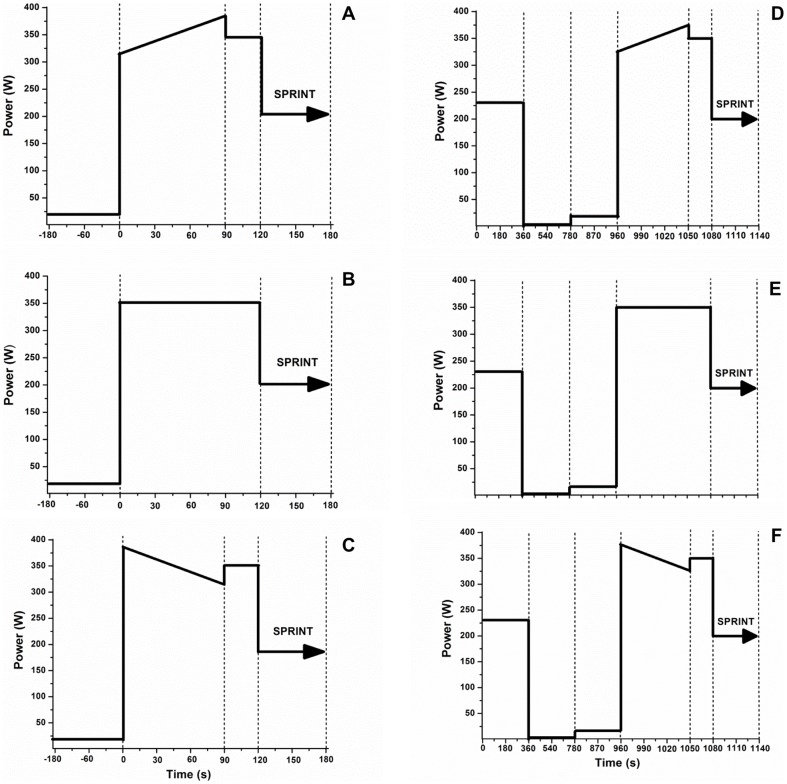
Study design for the six separate exercises conditions for a representative individual. Panels A, B and C - Paced exercises in the control condition, using slow start, even start and fast start, respectively. Panels D, E and F - Paced exercise preceded by previous heavy exercise (PHE), using slow start, even start and fast start, respectively.

The exercise protocol began with a 5 min warm-up at lactate threshold, followed by 7 min of rest. Thereafter, the subjects performed 3 min at 20 W before the experimental conditions. In the even-start (ES) condition, the athletes performed 2 min of constant-load exercise at P3-min, followed by a 1-min all-out exercise period. In the fast-start (FS) condition, the first 90 s of exercise was performed as the work rate was reduced linearly from 110% to 90% of P3-min, followed by a 1-min all-out exercise period. In the slow-start (SS) condition, the first 90 s of exercise was performed as the work rate was increased linearly from 90% to 110% of P3-min, followed by a 1-min all-out exercise period. The last 30 s of the FS and SS conditions was performed at P3-min [5]. During the first 2 min of exercise, a hyperbolic mode was used (fixed power), which was immediately changed to a linear mode (power dependent on the cadence) during the all-out exercise. These experimental conditions were performed in random order with and without previous exercise (Figure 1).

### 1-min all-out exercise

Following the 2-min pacing exercises, the athletes performed 1 min of all-out exercise. They were required to reach the peak power as quickly as possible and to exert maximal effort during the whole test. Throughout the 1-min test, the athletes were given verbal encouragement but were not informed of time elapsed. The VO_2_ was measured breath-by-breath during the exercise, and the data were reduced to 15-s stationary averages. The resistance to pedaling was calculated using the preferred cadence obtained during the incremental test and the workload corresponding to 50%Δ:
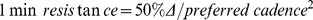
(1)


The following performance data were obtained from the all-out test: peak power output, time to peak power output and mean power output.

### Prior exercise

The prior exercise conditions involved participants performing 3 min of baseline cycling at 20 W, followed by a square-wave transition to a work rate requiring 90% CP (i.e., heavy-intensity exercise). At 6 min, the subjects were allowed to “spin down” against zero resistance for 1 min and then rested passively for 6 min before remounting the ergometer and pedaling for 3 min at 20 W. After this 3-min period, one of the three pacing conditions was immediately imposed as described above. One minute before and immediately after these exercise bouts, a fingertip capillary blood sample was taken to determine the blood [La]. The subjects repeated this process on separate days and in a randomized order until all experimental trials were completed.

### VO_2_ kinetics

The breath-by-breath data from each exercise test were filtered manually to remove outlying breaths, which were defined as breaths deviating by more than four standard deviations from the preceding five breaths. The breath-by-breath data were subsequently linearly interpolated to provide second-by-second values and aligned by time to the start of the exercise, and a nonlinear least squares algorithm was used to fit the data thereafter. A single-exponential model without a time delay and with a fitting window commencing at t = 0 s (equivalent to the mean response time) was used to characterize the kinetics of the overall VO_2_ response during initial phase (i.e., 90 s) of the different pacing strategies for all subjects. The following equation describes this model:
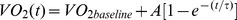
(2)


where VO_2_(t) represents the absolute VO_2_ at a given time t, VO_2_baseline represents the mean VO_2_ measured over the final 60 s of baseline pedaling, and A and τ represent the amplitude and time constant, respectively, which describe the overall increase in VO_2_ above the baseline. The oxygen deficit was also calculated for the same time period (i.e., 90 s) by multiplying the mean response time and the ΔVO_2_.

### Statistical analysis

The data are reported as the means ±SD. The normality of data was checked by the Shapiro-Wilk test. The data were analyzed using two-way ANOVA (prior exercise *vs*. pacing strategy), with Fisher's LSD test where appropriate. For all statistics, the significance level was set at p≤0.05.

## Results

During the ramped incremental test, the subjects attained a peak work rate (i.e., PVO_2_max) of 411±45 W, a VO_2_max of 4.43±0.47 L.min^−1^, a peak [La] of 8.6±1.6 mM and a maximal heart rate of 193±8 bpm. The CP and the W′ were 283±35 W and 22±6 kJ, respectively. The P3-min was calculated to be 407±47 W. The goodness-of-fit of the power-time relationship was R^2^ = 0.98. The SEE of the CP estimation was 7.0±6.6 W.

**Figure 2:**
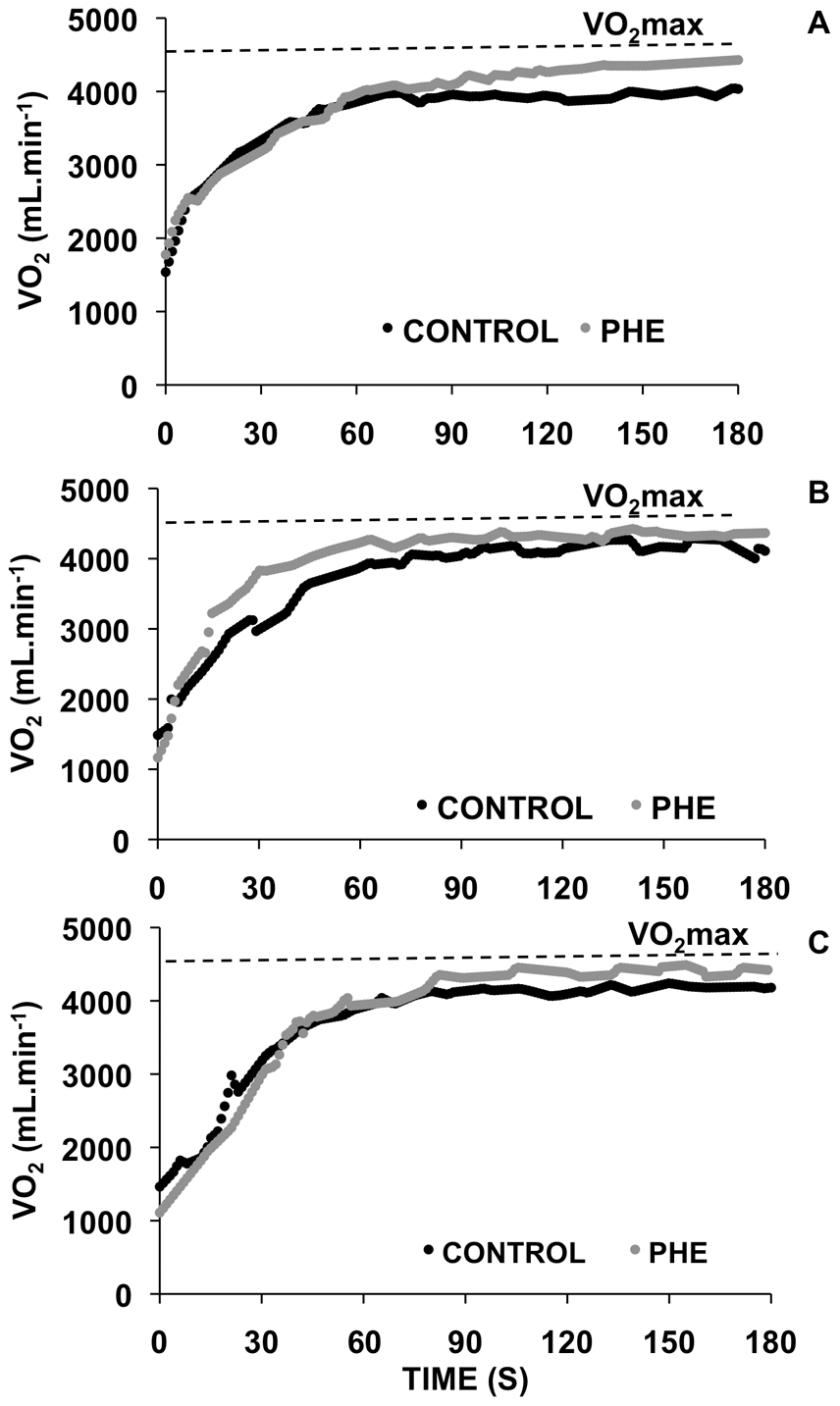
Oxygen uptake (VO_2_) responses during the pacing exercise conditions in a representative subject. The horizontal line superimposed on each panel indicates the subject's VO_2_max. Panels A, B and C - slow start, even start and fast start pacing conditions, respectively. Grey circles and black circles - paced exercise trials, with and without prior exercise, respectively. Notice that the VO_2_ response is speeded using the fast start pacing strategy only in the control condition. Thus, the fast start pacing strategy does not magnify the positive effects of prior heavy-intensity exercise on overall VO_2_ response.

The parameters of the VO_2_ kinetics during the paced exercise trials (FS, ES and SS) with and without prior exercise are presented in table 1. The measurements of VO_2_ amplitude (i.e., A) revealed a main effect of prior exercise (F = 37.95; p<0.001), but no interaction was detected (F = 1.62; p = 0.212). Similarly, the absolute VO_2_ (i.e., A+ VO_2_baseline) of the pacing exercises revealed a main effect of prior exercise, but no interaction was detected (F = 0.81; p = 0.452). The analysis of the O_2_ deficit values revealed a significant interaction (F = 3.95; p = 0.028), indicating that the effect from previous exercise occurred only for the ES and SS conditions. Post hoc analyses revealed a significant reduction in the O_2_ deficit only for the SS (p = 0.003) and ES (p<0.001) conditions after prior exercise. The effect of the pacing strategy was only significant when comparing SS with FS (p = 0.002) and ES with FS (p<0.001) during the control condition. The analysis of the mean response time values revealed a significant interaction (F = 3.59; p = 0.037), indicating that the effect of previous exercise occurred only for the ES and SS conditions. Post hoc analyses revealed a significant reduction in mean response time for the SS (p<0.001) and ES (p<0.001) conditions after prior exercise. The effect of the pacing strategy was only significant when comparing SS with FS (p<0.001) and ES with FS (p<0.001) in the control condition. Figure 2 shows the VO_2_ responses during the paced exercise trials (FS, ES and SS) with and without prior exercise in a representative subject.

**Table 1:** Parameters of the oxygen uptake (VO_2_) kinetics during paced exercise trials (FS, ES and SS), with and without prior exercise.

	Control			After heavy exercise			Significance
	FS	ES	SS	FS	ES	SS	
VO_2b_ (L.min^−1^)	1.27	1.26	1.20	1.13	1.26	1.13	NS
	0.20	0.21	0.22	0.22	0.25	0.23	
A (L.min^−1^)	2.67	2.73	2.63	2.89	2.86	2.91	*F = 37.95
	0.31	0.40	0.36	0.42	0.42	0.40	p<0.001
Absolute VO_2_ (L.min^−1^)	3.94	3.99	3.82	4.03	4.12	4.04	*F = 12.86
	0.31	0.36	0.33	0.39	0.31	0.34	p = 0.001
MRT (s)	27	32	32	24	25	26	†F = 3.59
	4	5•	6•	5	5‡	5‡	P = 0.037
CI 95 (s)	2.2	2.2	2.5	1.5	1.9	2.1	-
	0.7	0.5	0.8	0.6	0.7	0.7	
O_2_ deficit (L)	1.22	1.48	1.45	1.16	1.15	1.22	†F = 3.95
	0.25	0.27•	0.38•	0.40	0.25‡	0.30‡	P = 0.028

Data are the mean +SD. N = 14.

VO_2b_, baseline oxygen uptake; A, amplitude; Absolute VO_2_, VO_2b_+A; MRT, mean response time; CI 95, 95% confidence interval for MRT estimation. FS, fast start; ES, even start; SS, slow start.

*Main effect of previous exercise;

† Prior *vs*. pacing interaction;

‡ p<0.05 relative to the control condition;

• p<0.05 relative to the FS condition.

The VO_2_peak attained during the sprint for the FS, ES and SS conditions was significantly lower than VO_2_max, both with (FS = 4.19±0.35, ES = 4.14±0.33 and SS = 4.03±0.32 L.min^−1^; F = 5.678, p = 0.002) and without (FS = 3.90±0.32, ES = 4.01±0.30 and SS = 3.99±0.73 L.min^−1^; F = 5.678, p = 0.002) a prior exercise session. The VO_2_peak attained during the sprint for the FS, ES and SS conditions was unaffected by the prior exercise and pacing strategies (p>0.05).

**Figure 3:**
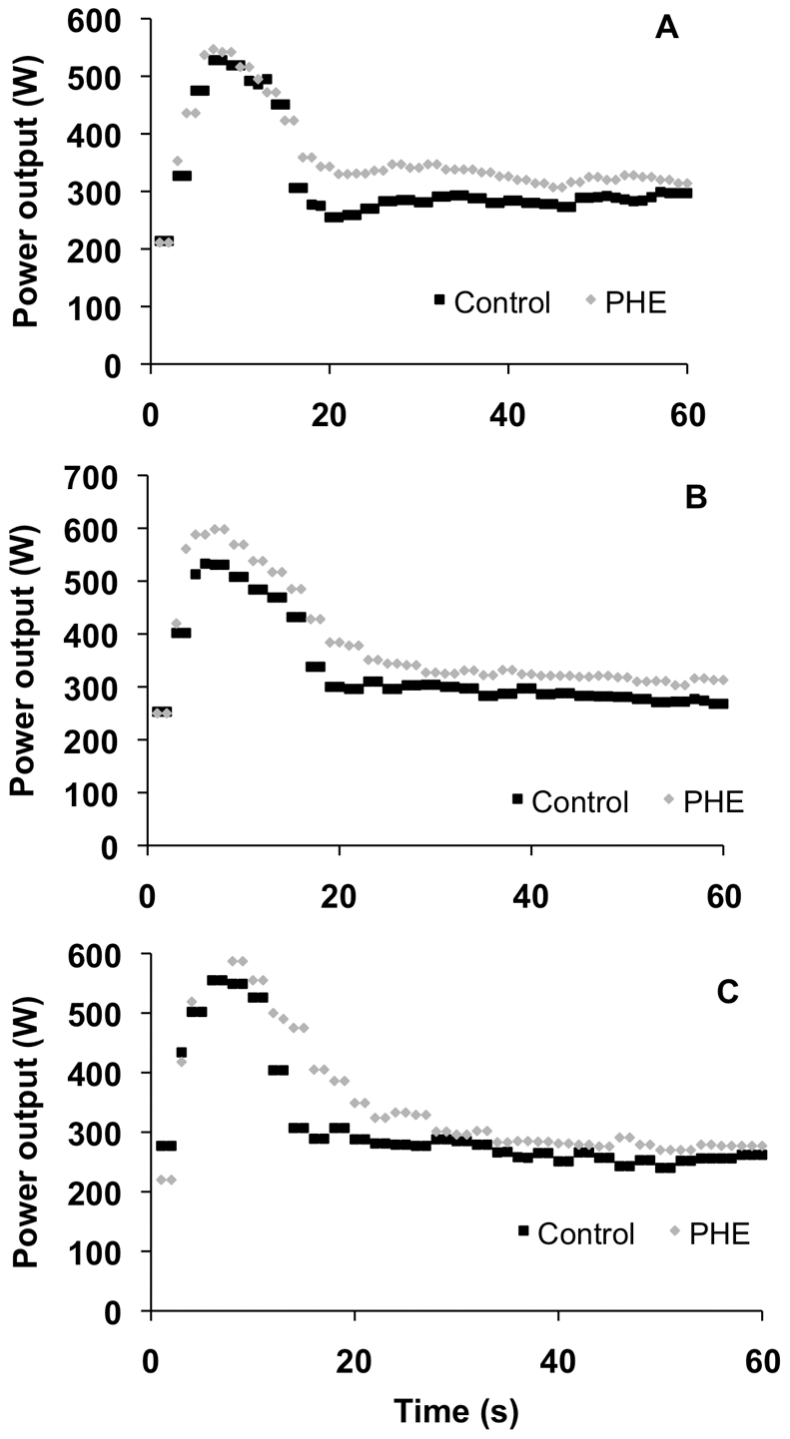
Power output during the pacing exercise conditions in a representative subject. Panels A, B and C - slow start, even start and fast start pacing conditions, respectively. Grey lozenges and black squares - paced exercise trials, with and without prior exercise, respectively. Notice the effect of prior exercise on performance, irrespectively of the pacing strategy used.

The parameters of exercise performance during the paced exercise trials (FS, ES and SS) with and without prior exercise are presented in table 2. The measurements of peak power output revealed a main effect of prior exercise (F = 61.72; p<0.001), but no interaction was detected (F = 2.28; p = 0.116). Similarly, the measurements of mean power output revealed a main effect of prior exercise (F = 6.54; p = 0.015), but no interaction was detected (F = 0.14; p = 0.873). The analysis of the time to peak power output values revealed no significant interaction (F = 0.52; p = 0.598), and no significant main effect of prior exercise (F = 0.59; p = 0.447) and pacing strategy (F = 2.38; p = 0.106). Figure 3 shows the power output during the paced exercise trials (FS, ES and SS) with and without prior exercise in a representative subject.

**Table 2:** Parameters of exercise performance during paced exercise trials (FS, ES and SS), with and without prior exercise.

	Control			After heavy exercise			Significance
	FS	ES	SS	FS	ES	SS	
PP (W)	606	558	553	674	617	586	*F = 61.72
	101	93	94	110	110	106	p<0.001
TPP (s)	6	6	7	6	6	7	NS
	2	2	3	1	1	1	
MPO (W)	400	396	386	415	408	396	*F = 6.54
	44	43	69	60	47	68	p = 0.015

Data are the mean +SD. N = 14.

PP, peak power output; TPP, time to peak power output; MPO, mean power output. FS, fast start; ES, even start; SS, slow start;

*Main effect of previous exercise.

The measurements of the [La] values before the paced exercises revealed a main effect of prior exercise (F = 58.77; p<0.001), but no interaction was detected (F = 0.01; p = 0.992). The mean [La] values after prior exercise for the FS, ES and SS conditions were 1.87±0.50, 1.78±0.61 and 1.73±0.74 mM, respectively. The mean control [La] values for the FS, ES and SS conditions were 1.14±0.41, 1.01±0.18 and 0.98±0.24 mM, respectively. The analysis of the [La] values after the sprint revealed no significant interaction (F = 2.00; p = 0.149), with no significant main effect of prior exercise (F = 0.79; p = 0.381) and pacing strategy (F = 1.15; p = 0.329). The mean [La] values after prior exercise for the FS, ES and SS conditions were 11.0±1.98, 11.1±2.39 and 10.1±2.32 mM, respectively. The mean control [La] values for the FS, ES and SS conditions were 11.3±3.11, 9.46±2.15 and 10.2±2.28 mM, respectively.

## Discussion

The purpose of this study was to determine the independent and additive effects of prior heavy-intensity exercise and pacing strategies on VO_2_ kinetics and performance during high-intensity exercise. Similar to previous studies, we have demonstrated that both priming exercise [6] and pacing strategies (i.e., FS) [5], [7] accelerated the overall VO_2_ kinetics (i.e., mean response time). However, our study reveals, for the first time, that an FS pacing strategy does not magnify the positive effects of prior heavy-intensity exercise on the overall VO_2_ kinetics. Moreover, the performance during high-intensity exercise (i.e., peak power output and mean power output) was enhanced only by prior heavy-intensity exercise. These data confirm and extend the proposal that the changes (i.e., speeding/slowing) in the VO_2_ kinetics during the initial phase of different pacing strategies (FS, ES and SS) are not necessarily associated with the changes in performance during short-term high-intensity exercise [5].

Some studies have found that the overall VO_2_ kinetics is accelerated by an FS pacing strategy when compared with ES and SS strategies [5], [7]. Factors such as exercise modality [5], [11] and aerobic performance level [6] do not appear to influence the effects of the FS pacing strategy on the overall VO_2_ kinetics. Thus, our data confirm that an FS pacing strategy can improve the overall VO_2_ kinetics during high-intensity exercise in trained endurance cyclists. Studies have shown a direct proportionality between the products of PCr splitting and muscle or pulmonary VO_2_ [19]. An FS pacing strategy requires a greater initial rate of muscle ATP hydrolysis, resulting in a greater initial Δ [PCr]/Δ time. Thus, a more rapid accumulation of the metabolites (ADP, Pi and Ca^2+^) that stimulate oxidative phosphorylation would be observed during an FS pacing strategy. Accordingly, Bailey et al. [5] used near-infrared spectroscopy (NIRS) to verify that FS strategies might be linked to increased muscle O_2_ extraction.

The classic experiments of Gerbino et al. [16] demonstrated that prior heavy exercise accelerated the monoexponential kinetics (i.e., mean response time) during a second bout of heavy exercise performed 6 min after the first. Later, studies using different experimental designs (e.g., intensities, durations of recovery time and age group) [6], [25] confirmed the seminal results obtained by Gerbino et al. [16]. Our experimental results revealed that both the mean response time and VO_2_ amplitude (the overall increase in VO_2_ above the baseline) were modified by previous heavy exercise. The increased VO_2_ amplitude during a second bout of severe exercise has been considered important for exercise tolerance/performance, because the slow component of the VO_2_ kinetics, the change in blood lactate concentration and the aerobic contribution are positively modified during the second bout of exercise. Central (increases in bulk O_2_ delivery) and peripheral (convective O_2_ delivery and increased activity of mitochondrial enzymes) factors are possible explanations for this altered VO_2_ response profile during the second bout of exercise [15].

To the best of our knowledge, this study is the first to determine the possible additive effects of priming exercise and pacing strategy on VO_2_ kinetics during severe-intensity exercise. We have demonstrated that previous heavy-intensity exercise accelerated the overall VO_2_ kinetics only during SS and ES pacing strategies. Moreover, there was no significant difference among the FS, ES and SS preceded by previous heavy-intensity exercise. Thus, the effects of priming exercise on VO_2_ kinetics during severe-intensity exercise are dependent on pacing strategy. Moreover, these effects do not appear to be magnified by an FS pacing strategy. Together, these results suggest that previous exercise has great potential to enhance the overall VO_2_ kinetics and that an FS pacing strategy does not amplify its effects.

In the present study, we did not use a biexponential model to characterize the VO_2_ kinetics because we were unable to repeat each trial to enhance the signal-to-noise ratio of the VO_2_ responses (see below). Thus, the parameters of the VO_2_ kinetics were not characterized. However, the exercise intensity used during the different pacing strategies was similar to PVO_2_max; therefore, the VO_2_ slow component, that elevates the VO_2_ above the steady-state value predicted from the sub-lactate threshold VO_2_-work rate relationship [26], cannot be detected under these circumstances. Thus, in line with other studies, the previous exercise may have only enhanced the VO_2_ amplitude [15], while the pacing strategy enhanced the time constant of the primary component of the VO_2_ response [5], [7]. Nevertheless, previous heavy-intensity exercise blunted the effects of the FS pacing strategy on the overall VO_2_ kinetics. Therefore, the alterations caused by previous exercise (available O_2_, convective O_2_ delivery, activity of mitochondrial enzymes and motor unit recruitment) appear to prevent the effects of an FS pacing strategy on the VO_2_ response. In line with this statement, Rossiter et al. [27] have found that prior high-intensity exercise reduced the amplitude of the [PCr] response, with the initial rate of [PCr] change (d[PCr]/dt) remaining unaffected during a second bout of heavy exercise. These alterations are suggestive of a reduced τ[PCr] during primed exercise [27], although the difference between conditions (i.e., control, 34 s *vs*. primed exercise, 32 s) did not reach statistical significance. Moreover, it has been demonstrated that the intramuscular enzyme activity status (i.e., pyruvate dehydrogenase complex - PDC), can allow a greater flux of acetyl groups into the mitochondria for oxidation [28]. An increased activation of pyruvate dehydrogenase complex might reduce both substrate-level phosphorylation (i.e., glycolysis and the creatine kinase and adenylate kinase reactions) [28] and the primary component time constant [29]. Thus, these mechanisms (altered [PCr] kinetics and/or increased PDC activity) might have blunted the effects of an FS pacing strategy on the VO_2_ response (i.e., VO_2_ “error signal”). However, future studies, with appropriated experimental design, should be conducted to confirm (or not) these hypotheses.

Previous heavy- or severe-intensity exercises have been shown to improve exercise tolerance during both submaximal [6] and perimaximal exercise [14]. This positive effect seems to be influenced by an optimal interaction between prior exercise intensity and recovery duration [6]. We have provided the first demonstration that prior heavy-intensity exercise enhances performance (peak power output and mean power output), and this increase occurs independently of the chosen pacing strategy. Improved exercise tolerance during submaximal intensity has been observed when the amplitude of the slow component of the VO_2_ kinetics decreased and the overall VO_2_ kinetics were faster [6]. Indeed, we have observed that the overall VO_2_ kinetics was faster and the VO_2_ amplitude increased (and thus the magnitude of the O_2_ deficit was reduced) after heavy-intensity exercise. Thus, previous heavy-intensity exercise can reduce the W′ utilization during the initial phase of exercise, improving performance during short-term high-intensity exercise.

It has been proposed that the mild lactic acidosis caused by prior heavy exercise might increase oxygen delivery by stimulating vasodilatation and a rightward shift in the oxyhaemoglobin dissociation curve (i.e. the Bohr effect) [16]. Based on data obtained in active subjects (VO_2_max∼50 mL.kg^−1^.min^−1^), some studies have suggested that a baseline blood lactate concentration of ∼3 mM results in an increased time to exhaustion during subsequent high-intensity exercise [6], [14]. Moderate-intensity prior exercise, which did not alter the baseline blood lactate concentration, does not enhance VO_2_ kinetics or exercise tolerance during subsequent high-intensity exercise performed by active subjects [16]. Similarly, Bailey et al. [6] have shown that the effect of prior heavy exercise on VO_2_ kinetics is prevented when baseline blood [lactate] recovers to <2 mM. However, we have verified that a baseline blood lactate concentration of ∼1.8 mM has enhanced both overall VO_2_ kinetics and short-term high-intensity performance in trained endurance cyclists. The low blood lactate concentration found 9 min after heavy intensity exercise can be explained, at least in part, by increased rate of blood lactate removal found in aerobic trained athletes [30]. Interestingly, Burnley et al. [31] have found that moderate-intensity prior exercise enhanced both primary VO_2_ amplitude and exercise performance in well-trained cyclists (VO_2_max ∼58 mL.kg^−1^.min^−1^). Thus, in aerobic trained cyclists, it seems that the presence of an elevated blood lactate concentration is not a *sine qua non* condition for improving VO_2_ kinetics and short-term high-intensity performance after prior exercise.

Some studies found that an FS pacing strategy can improve exercise tolerance [7] and performance [5] during short-term high-intensity exercise. In the present study, the pacing strategy did not significantly influence the exercise performance, although the overall VO_2_ kinetics was improved by the FS pacing strategy. Some interventions (priming exercise and pacing) have shown similar results [5], [6], indicating that changes in the overall VO_2_ kinetics will not necessarily enhance exercise tolerance/performance during subsequent high-intensity exercise. Interestingly, Bailey et al. [5] reported that utilizing an FS pacing strategy with active individuals (VO_2_max ∼52 mL.kg^−1^.min^−1^) improved both the overall VO_2_ kinetics and exercise performance during subsequent high-intensity exercise. In the present study, we analyzed trained endurance cyclists (VO_2_max = 62 mL.kg^−1^.min^−1^). Thus, differences in aerobic fitness might explain, at least in part, these different results. Bailey et al. [5] proposed that the attainment of VO_2_max during high-intensity exercise bouts, when this is ordinarily not possible, is essential for improving exercise performance. Given the finite speed of the VO_2_ response, the exercise durations at the extreme domain [32] would be too short to permit attainment of VO_2_max [21]. Thus, the attainment of VO_2_max would allow a more complete depletion of W′ and consequently allow better exercise performance [5]. Indeed, we have verified that VO_2_max was not attained during the FS pacing strategy. However, future studies using different experimental designs should be conducted to test this relationship.

Because of the nature of the present experiments, certain limitations of the study should be considered when interpreting its findings. The determination of the VO_2_ response parameters in the heavy- and severe-intensity domain using only one transition can have potential limitations (i.e., low confidence in the response parameters). Repeated bouts have traditionally been averaged to improve the signal-to-noise ratio of data [33]. However, due to the extremely demanding nature of the exercise testing and the frequent laboratory visits (11), only one trial was conducted for each experimental condition. Although we only measured one transition, the signal-to-noise ratio of the data can be improved by using higher VO_2_ amplitudes [33]. Therefore, higher VO_2_ amplitudes, as utilized in the present study, correspond to smaller confidence intervals. Indeed, the 95% confidence interval for the estimation of mean response time was <3 s for all conditions (Table 1).

In summary, we have demonstrated in trained endurance cyclists that priming heavy-intensity exercise has a positive effect on both overall VO_2_ kinetics and short-term high-intensity performance. However, the FS pacing strategy only modified the overall VO_2_ kinetics. This finding suggests that faster overall VO_2_ kinetics does not, *per se*, determine the performance (i.e., peak power output and mean power output) during high-intensity exercise. The FS pacing strategy does not magnify the positive effects of prior heavy-intensity exercise on the overall VO_2_ kinetics. Thus, the modifications caused by priming exercise preclude the effects of the FS pacing strategy on the overall VO_2_ kinetics. Finally, priming exercise seems to have greater potential than FS pacing strategies to enhance both overall VO_2_ kinetics and short-term high-intensity performance in trained endurance cyclists.
